# Control of CAR-T cell activity in space and time: the next level of anti-tumor action

**DOI:** 10.1038/s41392-023-01424-5

**Published:** 2023-04-10

**Authors:** Jessica Hoppstädter, Alexandra K. Kiemer

**Affiliations:** Department of Pharmacy, Pharmaceutical Biology, Saarland University, Saarbrücken, Germany

**Keywords:** Tumour immunology, Tumour immunology

In two recent papers published in Science, advanced synthetic biology techniques were used to overcome the limitations of chimeric antigen receptor (CAR)-T cell therapy: In one case, CAR-T cells were successfully introduced into and maintained in solid tumors,^1^ and in another approach, CAR-T cell proliferation and activation could be turned on and off in a time-dependent manner by drug administration.^2^ These approaches might improve therapy for a large number of solid malignancies.

Cell-based therapies using CAR T cells have introduced synthetic biology into the clinic to treat various types of blood cancers. The patient’s T cells are engineered with a synthetic receptor, i.e., the CAR, that helps recognize and attack cancer cells. The genetically modified T cells are then reinfused into the patient’s body, and a significant proportion of patients have been shown to be cured of difficult-to-treat cancers. Nevertheless, there are several limitations: On the one hand, the therapy can cause significant collateral damage by inducing a cytokine storm and attacking normal, non-cancerous cells. On the other hand, the efficiency of CAR-T cells may decrease over time, a phenomenon linked to a process denoted as T cell exhaustion.^3^

While CAR-T cells have shown impressive success in hematological cancers, solid tumors represent the majority of malignancies in clinical practice. They often harbor an immunosuppressive tumor microenvironment (TME), which prohibits the success of CAR-T cell-based therapies. In contrast to hematological malignancies, solid tumors also raise the problem of finding the ideal target antigen: Tumor-associated antigens overexpressed in solid tumors are expressed in many human body tissues, albeit to a lesser extent. Without tumor antigen specificity, the risk of significant on-target off-tumor toxicity increases substantially.^4^

**Fig. 1:**
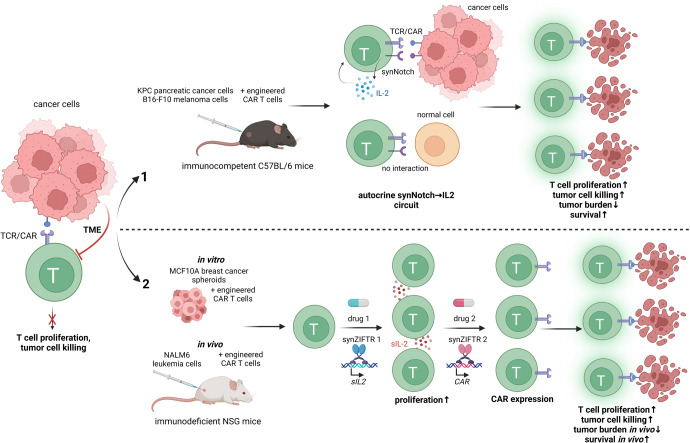
Two novel synthetic biology approaches aim to overcome present limitations of CAR-T cell therapy. Upper panel (1): To overcome tumor microenvironment (TME) suppression of CAR-T cells in solid malignancies, Allen et al.^1^ developed circuits in which tumor-specific synthetic Notch (synNotch) receptors locally induced the production of IL-2 trigged by tumor antigens independent from TCR/CAR activation, thereby restricting IL-2 secretion to the tumor site and bypassing the suppressive effects of the TME. Subsequently, autocrine IL-2 signalling promoted CAR-T cell proliferation. This approach increased CAR-T cell infiltration and tumor clearance in immunocompetent models of pancreatic cancer and melanoma. Lower panel (2): Li et al.^2^ developed compact synthetic zinc finger transcriptional regulators (synZiFTRs) to control the transcription of therapeutically relevant genes in CAR-T cells using FDA-approved small molecule inducers. In this proof-of-principle study, it was shown that it is possible to instruct T cells by the use of such drugs to sequentially activate multiple cellular programs such as proliferation and antitumor activity. Studies performed in vitro in breast cancer cells and in vivo in immunodeficient mice showed that a sequential induction of superIL-2 (sIL-2, an enhanced version of IL-2 with stronger IL-2 receptor affinity) and CAR can be beneficial for the anti-tumor effect. Created with BioRender.com

To overcome the immunosuppressant environment in malignant diseases, recombinant cytokines, such as interleukin (IL)-2, which is essential for T cell proliferation and activation, can be administered. However, cytokine administration can induce severe systemic side effects resembling the clinical manifestation of septic shock and including symptoms such as hypotension, supraventricular tachycardia, or acute respiratory distress syndrome. Accordingly, local production of IL-2 to promote T cell proliferation and activity would be desirable (Fig. 1).

In order to make sure that T cells are activated within solid tumors and only there, Allen et al.^1^ employed modular synthetic Notch receptors (synNotch). Modular synNotch receptors were first described in 2016 as general systems that allow customized sensing/response behaviors and, in particular, are able to drive T cell cytokine profiles.^5^

By coupling TCR/CAR activation and synNotch-driven IL-2 production in an autocrine synNotch→IL-2 circuit, Allen et al.^1^ have now been able to selectively propagate the therapeutic T cell population within solid tumors, as shown in the syngeneic KPC pancreatic cancer model in immunocompetent C57BL/6 mice. Unlike in immunodeficient mice, a paracrine approach in which IL-2 and CAR were expressed in separate cell populations was ineffective in these animals. The authors speculated that autocrine cells have preferential access to self-produced IL-2, which is particularly important in environments with competing IL-2-consuming cells (e.g., Tregs, naive T cells, and other cells within the TME), also referred to as IL-2 sinks, that are present only in the immunocompetent model. The study also showed that constitutive or TCR/CAR-activation-induced IL-2 expression in CAR-T cells was less efficient, most likely due to reduced T cell viability and the persisting sensitivity to the suppressive TME, respectively. Of note, expressing the autocrine synNotch→IL-2 circuit in CAR T cells improved anti-tumor activity not only in the KPC but also in a B16-F10 melanoma model. Despite its potency, the treatment showed no evidence of systemic cytokine toxicity or exacerbation of CAR T-cell toxicity, as the required recognition of dual antigen inputs, i.e., CAR and synNotch antigens further increased the specificity of tumor targeting. In conclusion, combining a tumor-responsive TCR/CAR with an autocrine synNotch→IL-2 circuit results in a potent and localized anti-tumor response, which may prove extremely useful for the clinical use of CAR T cells.

In another approach, FDA-approved small molecules were used in order to regulate programmable orthogonal gene circuits.^2^ The advantage of this system is that the desired activities can be induced and titrated by different drugs at specific stages of therapy. This is realized by the use of zinc fingers (ZFs), i.e., small DNA binding domains that can be programmed to recognize new motifs. Activation of respective synthetic zinc finger transcription regulators (synZiFTR) can be regulated by safe compounds, such as the antiviral protease-inhibiting drug grazoprevir, the breast cancer drug 4-hydroxytamoxifen/tamoxifen, or the plant hormone abscisic acid. synZiFTRs can be employed to induce CAR expression but also to enhance CAR-T cell functions by supplying immunoregulatory cytokines, such as IL-2 and IL-12. With IL-2 representing an autocrine T cell mitogen, activating drugs might allow to switch T cell proliferation on and off. In addition, cytokines, such as IL-12, which are typically produced by innate immune cells, can promote T cell activity.

The authors could prove orthogonal patterns of gene activation in a time-dependent manner: the proliferation of primary human T cells could be switched on before CAR expression was induced. The resulting dual-switch cells showed an enhanced cytotoxic activity against human HER2-positive MCF10A breast cancer cells in 3D spheroids. In vivo mouse experiments using a NALM6 leukemia model in NSG, i.e., immunodeficient mice showed that dual-switch cells are more effective than constitutive CAR-expressing cells. However, sequential production of IL-2 and CAR could lead to the loss of an advantage that activated CAR T cells have over IL-2 sinks: Allen et al.^1^ showed that after activation, CAR T cells upregulate CD25, a subunit of the IL2 receptor. Thus, this attractive novel switchable approach still has to be verified in an immunocompetent in vivo model, i.e., in the presence of potentially competing IL-2-consuming cells, and its suitability for the therapy of solid malignancies needs to be evaluated in vivo.

Possible future developments could combine these new technologies that allow controlled spatial and temporal activation of CAR T cells: on the one hand, proliferation and activation can be spatially restricted to tumors, and on the other hand, the expression of CAR, stimulatory cytokines, or additional innate immune system adjuvants, can be controlled in a titrated and time-dependent manner. Although further studies are needed to evaluate the feasibility of the presented systems in humans, the studies by Allen et al. and Li et al. represent significant steps for the development of advanced and individualized CAR-T cell therapies.
